# CCR5 Antagonist Maraviroc Inhibits Acute Exacerbation of Lung Inflammation Triggered by Influenza Virus in Cigarette Smoke-Exposed Mice

**DOI:** 10.3390/ph14070620

**Published:** 2021-06-28

**Authors:** Maximiliano Ruben Ferrero, Cristiana Couto Garcia, Marcella Dutra de Almeida, Jullian Torres Braz da Silva, Daniella Bianchi Reis Insuela, Tatiana Paula Teixeira Ferreira, Diego de Sá Coutinho, Carolina Trindade de Azevedo, Patrícia Machado Rodrigues e Silva, Marco Aurélio Martins

**Affiliations:** 1 Laboratory of Inflammation, Oswaldo Cruz Institute, Oswaldo Cruz Foundation (FIOCRUZ), Av. Brazil 4365, Manguinhos, Rio de Janeiro CEP.21045-900, Brazil; maximiliano-ferrero@hotmail.com (M.R.F.); marcella.vls2@gmail.com (M.D.d.A.); julliantbs@gmail.com (J.T.B.d.S.); dany.bianchiri@gmail.com (D.B.R.I.); tatiana.ferreira@fiocruz.br (T.P.T.F.); diego_dsc_@hotmail.com (D.d.S.C.); carolta@ioc.fiocruz.br (C.T.d.A.); patsilva1910@gmail.com (P.M.R.eS.); 2 Laboratory of Respiratory Virus and Measles, Oswaldo Cruz Institute, Oswaldo Cruz Foundation (FIOCRUZ), Rio de Janeiro CEP.21045-900, Brazil; cristiana.garcia@ioc.fiocruz.br

**Keywords:** chemokine receptor, chronic obstructive pulmonary disease, Influenza A (H1N1), Maraviroc

## Abstract

Influenza A virus (IAV) infection is a common cause of acute exacerbations of chronic obstructive pulmonary disease (AECOPD). Since macrophage inflammatory protein 1 α, a chemokine that acts through CC-chemokine receptor (CCR)-5, appears elevated in COPD patients’ airways, we evaluated whether CCR5 antagonist Maraviroc could inhibit the exacerbated lung inflammatory response noted after IAV H1N1 infection in mice exposed to cigarette smoke (Cs). C57BL/6 mice, subjected or not to Cs inhalation for 11 days, were infected with H1N1 at day 7. Maraviroc (10 mg/kg) or dexamethasone (1 mg/kg) were given in a therapeutic schedule, followed by the analyses of lung function, survival rate, and inflammatory changes. As compared to mice subjected to Cs or H1N1 alone, the insult combination significantly worsened airway obstruction, neutrophil infiltration in the airways, and the survival rate. All changes were sensitive to Maraviroc but not dexamethasone. Maraviroc also reduced the accumulation of neutrophils and macrophages as well as CXCL1 production in the lung tissue, and serum levels of IL-6, whereas comparable viral titers in the lungs were noted in all infected groups. Collectively, these findings suggest that Maraviroc oral treatment could be an effective therapy for controlling acute exacerbations of respiratory diseases such as COPD.

## 1. Introduction

Chronic obstructive pulmonary disease (COPD) is a major health problem with increasing prevalence, affecting 216 million people worldwide in 2016 [1]. COPD is marked by a not fully reversible airflow limitation, which is usually progressive and accounted for by lung inflammation [2]. Cigarette smoking is associated with 95% of COPD cases in industrialized countries, while other environmental pollutants are also important causes in developing countries [3].

Sustained pulmonary inflammation in response to cigarette smoke (Cs) is one of the most recognized causes of COPD pathogenesis leading to chronic bronchitis and emphysema as main clinical manifestations. Lungs of COPD patients present increased levels of pro-inflammatory mediators such as tumor necrosis factor-α (TNF-α), interleukin (IL)-8 and IL-1 among others, which mediate a constant influx of leukocytes into the airways, mainly neutrophils and macrophages [4]. Frequently, COPD patients experience acute exacerbations of symptoms (AECOPD) mostly triggered by infections. More than half of AECOPD is attributed to viral infections, and influenza A is among the more problematic due to the likelihood of epidemics and pandemics [5]. During AECOPD periods, the production of inflammatory cytokines and chemokines in the lung increases and so does leukocyte migration, with an exacerbated neutrophil activity. Consequently, AECOPD accelerates lung function deterioration, increases morbidity and mortality of COPD, and represents a major cause of COPD patients’ hospitalization [5,6]. Current pharmacological therapies, including glucocorticoids and bronchodilators, fail to control these exacerbation periods in part due to their low capacity to control non-type 2 pattern of inflammation [7,8,9]. This situation has a direct economic impact on the health system; as an example, the USA projected a budget of US$50 billion for COPD in 2010, from which $30 billion were destined to direct health care expenditures, principally hospitalizations [10].

Chemokines and their receptors play a key role in orchestrating and perpetuating airway inflammation in COPD. Particularly, Macrophage inflammatory protein 1 α (CCL3), a chemokine that acts through CC-chemokine receptor (CCR)-1 and CCR5, appears elevated in COPD patients’ airways [11]. Moreover, CCL3 stimulates IL-6 production in macrophages and there is a correlation between the augmented IL-6 levels in COPD patients’ serum with the higher occurrence of exacerbations and poor prognosis [12,13]. Furthermore, Cs enhances CCR5 expression in bronchial epithelium, and monocytes from COPD patients, but not from healthy smokers, which allows the increased migration in response to CCR5 ligands [14,15]. Despite CCR5 being strongly associated with the migration of macrophages and monocytes, it has been also implicated in the recruitment of neutrophils into the airways in two different models of acute lung injury [16,17]. Maraviroc is a CCR5 antagonist approved by the FDA in 2007 for the treatment of HIV infection that showed interesting anti-inflammatory properties in several mice models of lung or neuroinflammation [16,17,18]. In addition, it was recently reported that this drug is a high-affinity inhibitor of SARS-CoV-2 M^pro^ protease, a promising target for antiviral drug development [19].

AECOPD increases disease mortality and the demand for novel treatments to control these episodes is urgent. We thought to investigate the potential anti-inflammatory effect of the CCR5 antagonist Maraviroc in a mice model of acute exacerbation of lung inflammation, triggered by the combination of Influenza virus H1N1 infection and Cs exposure.

## 2. Results

### 2.1. Maraviroc Improves Lung Function and Survival in Mice Combining Cs Exposure and H1N1 Infection

**Figure 1:**
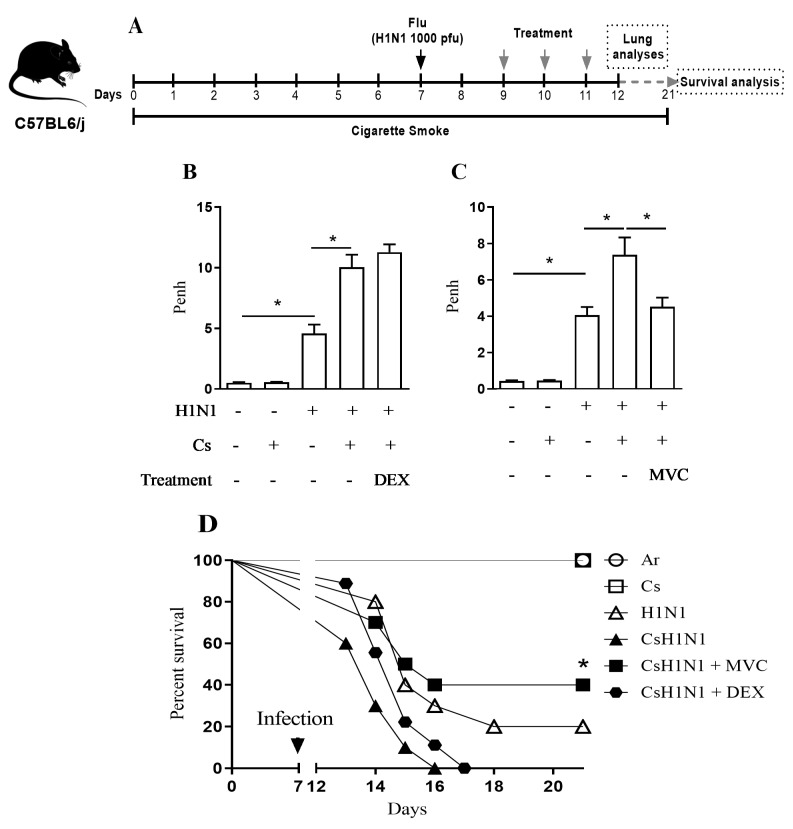
Maraviroc improves lung function and survival in mice, combining cigarette smoke exposure and H1N1 infection. Schematic representation of the experimental design (**A**). Effect of dexamethasone (DEX) (**B**) or Maraviroc (MVC) (**C**) on the exacerbation of enhanced pause (Penh) elevation in mice exposed to Cs and infected with influenza virus (CsH1N1)**.** Data are expressed as mean ± SEM from at least 6 mice per group. * for *p* < 0.05. Differences were statistically evaluated One-Way ANOVA with Tukey post-test. Survival proportions of the different groups of mice to day 14 after infection (**D**). *n* = 10 for each group. * for *p* < 0.05. Differences were statistically evaluated using Log-rank (Mantel–Cox) test.

Using the experimental protocol depicted in Figure 1A, we observed that all infected groups of mice showed a significant weight loss at the end of the experiment (Supplementary material (S) Figure S1). In comparison with mice only infected (H1N1) or with those only exposed to Cs, mice subjected to H1N1 infection and Cs exposure (CsH1N1) presented an exacerbated deterioration of lung function, measured 4 days post-infection (Figure 1B). Similar findings of exacerbation were observed concerning lethality evaluated at 6–15 days post-infection (Figure 1D), in line with our prior study [20]. Oral treatment with Maraviroc (10 mg/kg), at days 2, 3, and 4 post-infection, significantly improved both lung function deterioration (Figure 1C) and survival rate (Figure 1D) in CsH1N1 mice, while dexamethasone (1 mg/kg, oral) failed in both analysis (Figure 1B,D).

### 2.2. Maraviroc Reduces Neutrophil and Macrophage Infiltration in the Airways of Mice Experiencing Exacerbated Lung Inflammation by Combining Cs and Infection

**Figure 2:**
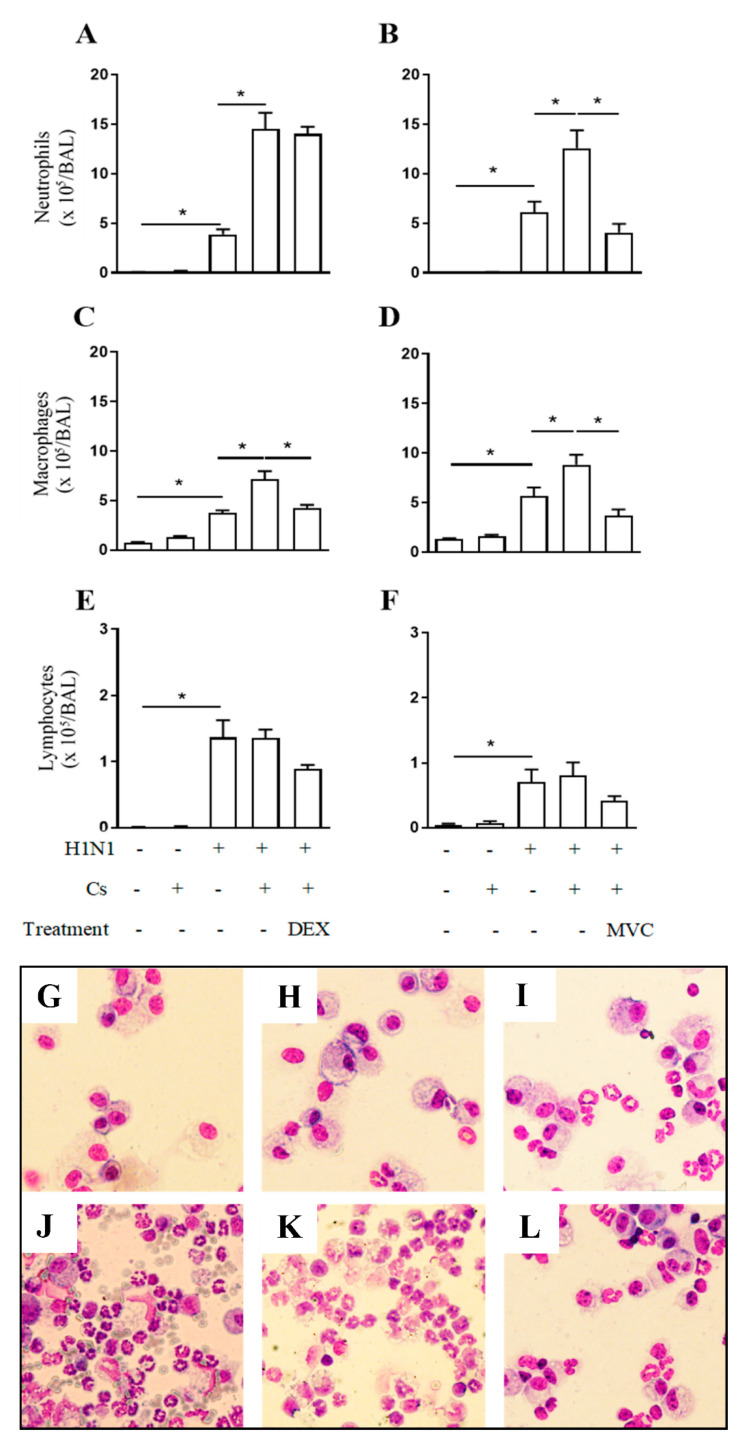
Maraviroc reduces neutrophil and macrophage infiltration in the airways of CSH1N1 mice. Effect of dexamethasone (DEX) or Maraviroc (MVC) on the BALF accumulation of neutrophils (**A**,**B**), macrophages (**C**,**D**), and lymphocytes (**E**,**F**) of mice exposed to Cs and infected with influenza A virus (CsH1N1). Data are expressed as mean ± SEM from at least 6 mice per group. * for *p* < 0.05. Differences were statistically evaluated One-Way ANOVA with Tukey post-test. Representative images (400× magnification) of cells recovered from BALF and stained with May-Grünwald Giemsa for Air (**G**), Cs (**H**), H1N1 (**I**), CsH1N1 (**J**), CsH1N1 + DEX (**K**), CsH1N1 + MVC (**L**) groups.

As compared to negative control mice, in atmospheric air conditions (Air), mice infected with H1N1 reacted with increased number of neutrophils (Figure 2A,B), macrophages (Figure 2C,D) and lymphocytes (Figure 2E,F) recovered in the BALF and revealed by May-Grunwald-Giemsa staining (Figure 2G–L). Although no significant changes were noted in the number of leukocytes in BALF samples from Cs mice, the number of neutrophils and macrophages, but not lymphocytes, recovered in BALF samples from CsH1N1 mice were significantly higher than those found in the H1N1 group (Figure 2A,D). Again, the interventional treatment with Maraviroc inhibited the upregulation of both neutrophil (Figure 2B) and macrophage numbers noted in CsH1N1 mice (Figure 2D) under conditions where dexamethasone was active against macrophages (Figure 2C) but not neutrophils (Figure 2A). None of the treatments reduced lymphocyte counts statistically in CsH1N1 mice (Figure 2E,F).

### 2.3. Maraviroc Reduces Increased Macrophage and Neutrophil Infiltration in the Lung Tissue under Combined Insult

**Figure 3:**
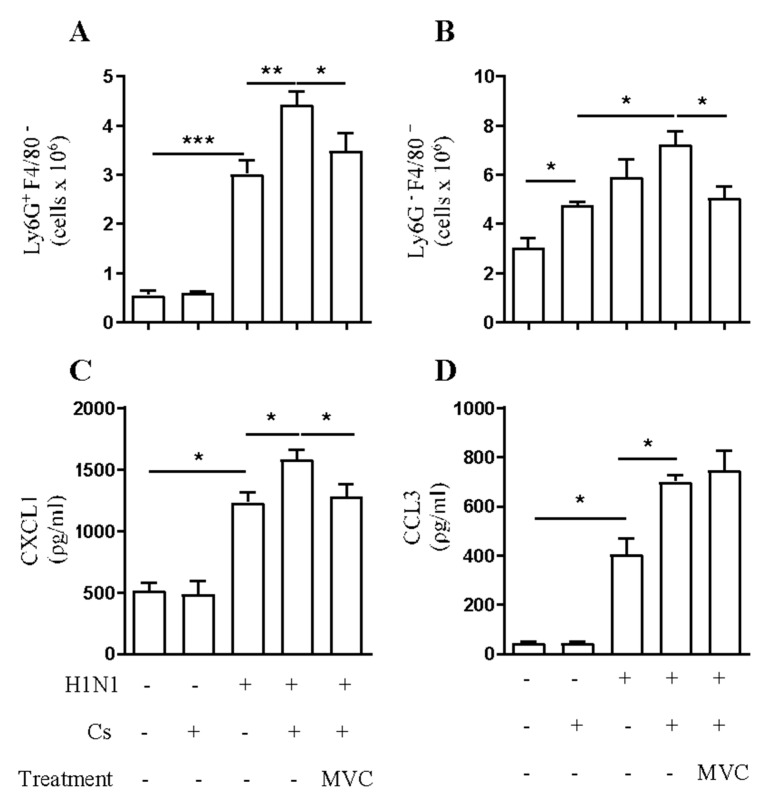
Maraviroc reduces increased macrophage and neutrophil infiltration in the lung tissue of CsH1N1 mice. Effect of Maraviroc on the numbers of (**A**) CD45^+^/F4/80^−^/Ly6G^+^ (Neutrophils), (**B**) CD45^+^/F4/80^+^/Ly6G^−^ cells (Macrophages), and the levels of CXCL1 (**C**) and CCL3 (**D**) in the lung tissue of mice exposed to Cs and infected with influenza A virus (CsH1N1). Data are expressed as mean ± SEM from at least 6 mice. * *p* < 0.05, ** *p* < 0.01, *** *p* < 0.001. Differences were statistically evaluated One-Way ANOVA with Tukey post-test.

We observed almost no neutrophils (CD45^+^/F480^−^/Ly6G^+^) in the lung tissue of the air group of mice by flow cytometry on day 12. Instead, viral infection induced a significant neutrophilic inflammation. Although there were no significant numbers of neutrophils in lung tissue of the Cs group, the combination of insults in CsH1N1 mice augmented neutrophil numbers by 50% compared to H1N1 mice (Figure 3A). On the other hand, macrophage population (CD45^+^/F480^+^/Ly6G^−^) in the lung tissue augmented 60%, 96%, and 140% in Cs, H1N1, and CsH1N1 mice, respectively, compared to control mice (Air) (Figure 3B). Maraviroc significantly reduced both neutrophil and macrophage numbers in the lung tissue of CsH1N1 mice by 22% and 30%, respectively (Figure 3A,B, respectively).

From the analysis of lung neutrophils (F480^−^/Ly6G^+^), we found that 30% of these cells were CCR5^+^ in the Air and the Cs group, while viral infection increased the percentage of CCR5^+^ neutrophils to 40% of the population in H1N1 and CsH1N1 groups (Figure S3A). In addition, 60% of macrophages (F4/80^+^/Ly6G^−^) were CCR5^+^ in control group. However, in Cs exposed mice, there was a mild increase in the percentage of CCR5^+^ macrophages compared to the Air group. Neither viral infection nor the combination of insults in CsH1N1 mice had significant impact on the percentage of CCR5^+^ macrophages in the lung tissue (Figure S3B).

As assessed by ELISA using lung tissue homogenates, viral infection provoked a 2.4-fold increase in lung CXCL1 compared to the Air group. Cs exposure alone did not induce a significant production of CXCL1. However, the combination of insults in the CsH1N1 group significantly increased CXCL1 production compared to the H1N1 group. Maraviroc treatment on CsH1N1 mice reduced CXCL1 production to levels observed in H1N1 mice (Figure 3C). In addition, we quantified CCL3, a known CCR5 ligand. H1N1 infection triggered a strong production of CCL3 compared to the Air group. While Cs mice showed no significant levels of CCL3 in lung tissue, the combination of insults in CsH1N1 mice exacerbated the production of this chemokine to levels significantly higher than those observed in H1N1 group. Treatment with Maraviroc did not affect CCL3 production in CsH1N1 mice (Figure 3D).

Chemokines can bind and activate more than one chemokine receptor. Particularly, CCL3 binds to and activates both CCR5 and CCR1 [21]. However, differently to what was observed with Maraviroc, the CCR1 antagonist J-113863 (10 mg/kg) did not improve lung function nor reduce leukocyte numbers in the airways of CsH1N1 mice (Figure S4).

### 2.4. Maraviroc Reduces IL-6 Levels in Mice Serum

**Figure 4:**
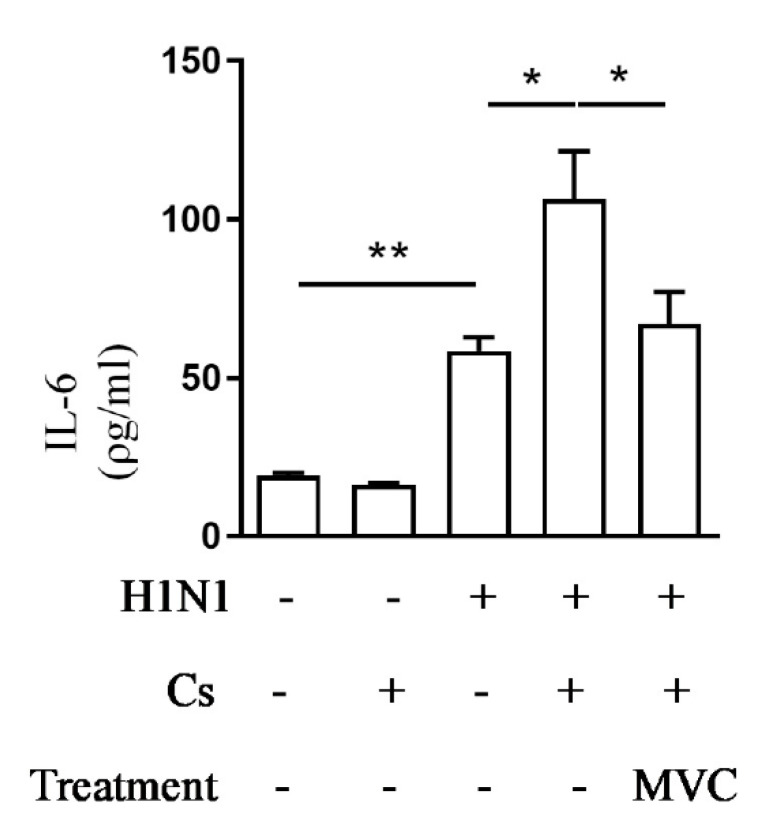
Maraviroc reduces IL-6 levels in mice serum. Effect of Maraviroc on IL-6 levels in the serum of mice exposed to Cs and infected with influenza A virus (CsH1N1). Data are expressed as mean ± SEM from at least 6 mice per group. * *p* < 0.05, ** *p* < 0.01. Differences were statistically evaluated One-Way ANOVA with Tukey post-test.

AECOPD periods are characterized by the rise of systemic inflammatory markers like IL-6 that correlates with worse patients’ outcomes [13]. In our model of experimental exacerbation, the systemic levels of IL-6 (serum samples) increased significantly on day twelve of assay in response to viral infection compared to the control Air group (Figure 4). As illustrated in the same figure, although in Cs mice there was no significant increase in the level of serum IL-6, the combination of insults (CsH1N1 group) led to significantly higher level of serum IL-6 than that observed in mice from the H1N1 group. Treatment with Maraviroc inhibited exacerbation of serum IL-6 levels in CsH1N1 mice (Figure 4).

### 2.5. Maraviroc Does Not Alter Viral Load in the Lung Tissue

While assessing whether or not the anti-inflammatory effect of Maraviroc is accounted for by an effect on viral load, we observed no significant difference between treated and untreated infected mice, as attested by the quantification of viral HA protein amount present in the lung tissue (Table 1).

## 3. Discussion

Viral and bacterial infections, as well as airway inflammation, are strongly associated with COPD severe exacerbations underlying what is called COPD crisis or AECOPD [22,23,24]. The understanding of the pathogenesis of exacerbations has been improved, but their clinical management remains problematic [23]. In this study, we assessed the therapeutic potential of the CCR5 antagonist Maraviroc to interfere with glucocorticoid-resistant exacerbation of lung inflammation in mice combining Cs exposure followed by H1N1 infection. Our findings revealed that the insult combination worsened both functional and pathological features of COPD, including acute deterioration of respiratory signals, lethality, neutrophilic infiltrate, and levels of systemic IL-6. All these changes were clearly sensitive to Maraviroc administered orally, but not dexamethasone, suggesting that the former has the potential to be repurposed as an effective therapy for controlling acute exacerbations by viral infections in diseases such as COPD.

Influenza A H1N1 is among the most frequently identified respiratory viruses associated with COPD exacerbations [22,24,25]. Prior studies have shown that the infection by H1N1 virus of mice subjected to Cs inhalation, for 12–20 days, can mimic the hyper-inflammation and other features of COPD exacerbation [20,26]. While the chemokine receptor CCR5 plays a deleterious role in different models of lung inflammation triggered by Cs [27,28], in models of H1N1 infection, CCR5 was shown to play a protective role [29,30]. Here, we have investigated whether the exacerbation of the lung inflammatory response, respiratory changes, and lethality seen in mice by combining Cs inhalation and H1N1 infection would be sensitive to the CCR5 antagonist Maraviroc.

The occurrence of AECOPD periods in patients is directly associated with lung function decline [31,32] and increased mortality risk [33]. We previously reported that the combination of Cs inhalation and H1N1 infection in our model induces an exacerbated decline of lung function also decreasing mice survival [20]. Here, we showed that the therapeutic treatment with Maraviroc for three days, prevented the aggravated decline of lung function presented in mice combining Cs exposure and viral infection, and also increased mice survival significantly.

Maraviroc treatment also presented a strong anti-inflammatory activity reducing the exacerbated influx of both neutrophils and macrophages towards the airways of CsH1N1 mice. Importantly, the reduction in leukocyte numbers by Maraviroc was not related to an anti-viral activity as all infected groups showed comparable viral titers in the lungs by the end of the experiments. On the other hand, dexamethasone treatment could only reduce macrophage numbers in the airways but not neutrophil numbers. Therefore, the exacerbated neutrophilic inflammation in our model appears to be corticosteroid resistant as it is observed in COPD patients [34].

As increased recruitment of leukocytes, especially neutrophils, to the airways has been extensively associated with lung function decline [35], our results suggest that the anti-inflammatory performance of CCR5 antagonism by Maraviroc improved lung function. Accordingly, it was previously reported that CCR5 blockade with specific antibodies reduced inflammation and improved lung function in a model of lung inflammation induced by IFN-γ [28]. Furthermore, in another COPD model induced by 4 or 24 weeks of Cs exposure, CCR5 absence also resulted in reduced inflammation without affecting airway remodeling [27].

In addition, treatment with Maraviroc reduced the numbers of macrophages and neutrophils in the lung tissue. Macrophages play an important role in the development of COPD and CCR5 is involved in the recruitment of monocytes from blood to the lung tissue in COPD patients [15,36]. Indeed, it is widely accepted that CCR5 is involved in monocyte chemotaxis. As showed by flow cytometry analysis of mice lung tissue, most of the CD45^+^F4/80^+^/Ly6G^−^ cells, identified as macrophages or monocytes [37], were CCR5^+^. Moreover, we observed a marked increase in the concentration of CCL3, a ligand of CCR5, in lung tissue of CsH1N1 mice compared to mice that were only infected. Then, it is very likely that treatment with Maraviroc, in our model, directly affected CCL3 signalization, reducing monocyte migration towards the site of injury, and macrophage accumulation into the lungs.

Even though neutrophil recruitment does not depend directly on CCR5 signaling, but on of ELR+ CXC chemokines receptors like CXCR1/2 [38,39], we found that influenza infection increased the percentage of CCR5^+^ neutrophils present in lung tissue as it was previously reported by Rudd JM, et al. [40]. In addition, Maraviroc treatment was able to reduce neutrophil recruitment in other mouse models of lung inflammation [16,17]. Here, we showed a strong inhibition of neutrophil recruitment towards lungs upon Maraviroc treatment, accompanied by a reduction in CXCL1 concentration. Since CXCL1 is a chemokine that induces neutrophil chemotaxis through CXCR1/2 and it is mainly synthesized by macrophages, among other cell types, the effect of Maraviroc over neutrophil accumulation in lungs of CsH1N1 mice could also be an indirect effect of the Maraviroc effect over macrophages.

There is limited evidence showing that both cigarette smoke and influenza infection can upregulate CCR1 expression [40,41]. In addition, few publications could show a role of CCR1 in lung injury using models of lung fibrosis [42,43], and as far as we know, there is just one publication showing an anti-inflammatory effect of CCR1 pharmacological blockade in a model of lung inflammation triggered by cigarette smoke [41]. On the other hand, as we are showing here, others also demonstrated a relevant anti-inflammatory effect of CCR5 pharmacological blockade in several models of lung injury in mice [16,17]. It is noteworthy that the CCR1 antagonist dose utilized in the study has been reported to be effective against experimental arthritis [44]. The possibility does exist that differently to what was observed in case of CCR5, the insult combination explored here is not followed by upregulation of CCR1 expression, which might help to explain the lack of relevance of this receptor in our model.

Some studies involving CCR5 KO mice have shown that the lack of CCR5 increases mice lethality and pathogenesis after influenza A infection, associated with reduced macrophage infiltration, but accumulation of neutrophils in the airways [29,30]. Here, we showed that targeting CCR5 after H1N1 infection, under CS exposure, is protective, reducing both macrophages and neutrophils in lungs and airways. Using a receptor antagonist in experimental models is a more feasible way to look for a therapeutic target to be translated to the clinics since knocking out a gene may cause compensatory circuits to take over the role of the missing gene [45]. Despite the different outcomes between the models and strategies, we have converged towards the same results regarding CCR5 lack of influence on viral loads [29].

Augmented levels of IL-6 in serum correlates with a state of systemic inflammation. In COPD patients, persistently elevated levels of serum IL-6 but not TNFα or IL-8 are associated with a higher frequency of exacerbations and poor clinical outcome [46]. Furthermore, augmented serum IL-6 level is predictive of increased mortality [46,47]. The combination of Cs and viral infection, in our model, increased serum IL-6 levels while it also reduced mice’s survival when compared to those that were only infected. Maraviroc could inhibit the exacerbation of serum IL-6 levels in CsH1N1 mice, also increasing mice survival while systemic administration of dexamethasone could not. Even though the combination of viral infection and Cs exposure to mimic AECOPD in mice was previously reported [26,48], as far as we know, the coexistence of exacerbated levels of serum IL-6 and reduced mice survival is a very unique feature of our model. This may be the consequence of using higher viral inoculum to infect mice compared to Bucher et al. [26], or a higher virulence of the Influenza virus strain used compared to that used by Oostwoud et al. [48].

Certain limitations exist when modeling human pathologies in animals. In our model, the reduced mice survival in a short period of time after infection limited the observation of chronic features of COPD like emphysema. At the same time, our model is not suitable to study the impact of exacerbations in the long term or how a certain treatment administrated during the exacerbation would affect outcomes in longer periods of time.

In conclusion, these findings demonstrate that the interventional treatment with Maraviroc given orally can neutralize exacerbation of lung inflammation and lethality in mice combining Cs and H1N1 infection, in a mechanism unrelated to the viral load. They also suggest that the CCR5 receptor is an appealing drug target concerning the management of virus-induced COPD exacerbations and excessive lung inflammation, as in cases of either Influenza A or SARS-Cov-2 infection.

## 4. Materials and Methods

### 4.1. Mice

Female C57/BL6 mice, 8–10 weeks old were obtained from the Institute of Sciences and Technology in Biomodels of the Oswaldo Cruz Foundation, Brazil. Mice were housed in ventilated cages in a controlled day-night cycle and had access to food and water ad libitum. All experiments were analyzed and approved by the local ethics committee: Ethical committee CEUA/IOC number LA-004/2017.

### 4.2. Cs-Induced Exacerbation of Influenza-A Infection Model

Female C57BL/6 mice weighing 18–20 g and 8–10 weeks old were exposed to Cs from 12 commercial full-flavored Marlboro cigarettes (10 mg tar, 0.9 mg nicotine, and 10 mg monoxide) per day or ambient air (controls) for 11 days, using a smoking chamber as described previously [49]. On the 7th day of exposure, animals were anesthetized with 50 µL *s.c.* of ketamine (60 mg/Kg) and xylazine (4 mg/Kg) solution and infected with 1000 pfu of influenza (H1N1) virus strain A/PR/8/34 H1N1 intranasally. On day 12 of Cs exposure, mice were euthanized with 100 µL *i.p.* of ketamine (300 mg/Kg) and xylazine (30 mg/Kg) solution and experiments were performed to evaluate lung inflammation. Alternatively, mice organized in the same experimental groups were evaluated during 14 days after H1N1 infection, maintaining Cs exposure, for lethality assessment. We accompanied mice weight loss and euthanized with 100-µL *i.p.* of ketamine (300 mg/Kg) and xylazine (30 mg/Kg) solution if they had lost more than 25% of the initial weight. The groups receiving only viral infection (H1N1), only Cs, or no insult (Air) were also included as controls.

### 4.3. Pharmacological Schedule

We used Maraviroc, a selective CCR5 antagonist, and J-113863, a selective CCR1 antagonist, from Tocris Bioscience (Bristol, UK) and dexamethasone from Sigma Chemical Co (St. Louis, MO, USA). Maraviroc (10 mg/Kg in saline solution), J-113863 (10 mg/Kg in saline solution), or dexamethasone (1 mg/Kg in saline solution) were administered by gavage once a day for 3 days starting at day 9, 48 h after the viral infection, 60 min before the first Cs exposure of the day.

### 4.4. Assessment of Pulmonary Mechanics

Non-invasive in vivo assessment of enhanced pause (Penh) was performed using barometric whole-body plethysmography (Buxco Research System, Wilmington, NC, USA) as we previously described [50]. Measurements were recorded for 5 min for each animal in conscious, spontaneously breathing mice. Penh values were obtained 24 h after the last Cs exposure on day 11.

### 4.5. Assessment of Leukocyte Content in the Airways

To assess the leukocytes in the airways, bronchoalveolar lavage fluid (BALF) was performed as we previously described [51]. Briefly, an incision was made in the trachea where a 1.7 mm outside diameter polyethylene catheter was inserted. Then, the airways were washed twice with 1mL of phosphate buffer saline (PBS) containing 10 mM ethylenediaminetetraacetic acid (EDTA). BALF was centrifuged (300× *g*, 10 min, 4 °C) and cell pellets were resuspended in 250 μL of PBS plus EDTA (10 mM). To quantify the leukocyte influx into the airway lumen, BAL effluent was diluted in Türk solution (2% acetic acid) and total leukocytes were counted in a Neubauer chamber, using a light microscope (BX40; Olympus, Center Valley, PA, USA). Differential cell counts were performed in cytospin smears stained with the May-Grünwald Giemsa method and analyzed using a light microscope (BX40, Olympus).

### 4.6. FACS Analyses of Lung Tissue

After the removal of the left lobe of mice lung, the tissue was chopped and digested with 15 mg/mL collagenase (Type D; Roche Diagnostics) and 25 μg/mL DNase (Type 1; Roche Diagnostics) in 4 mL RPMI + 10% CFS for 1 h at 37 °C with agitation. Lung was then passed through a 70 μm sieve (BD Bioscience). The resulting cell suspension was washed, red blood cells were lysed, and the remaining cells were resuspended in 1 mL RPMI. To analyze cell populations, the cell suspension was labeled using monoclonal antibodies anti-CD45 (clone 30-F11) (1:100) anti-Ly6G (clone 1A8) (1:400), anti-F4/80 (clone BM8) (1:100), anti-CCR5 (HM-CCR5 (7A4)) (1:200) from eBioscience for 30 min. All data was acquired by flow cytometry (FACSCalibur; BD Biosciences PharMingen) and analyzed using FlowJo software (Tree Star, Inc., Ashland, OR, USA). Gating strategies for the identification of neutrophils and macrophages in these experiments are shown in Figure S2.

### 4.7. Cytokine Analysis in Lung Tissue and Serum

After BALF, the right lung was removed and homogenized in 1 mL of PBS (0.4 M NaCl and 10 mM of NaPO_4_) containing anti-proteases (0.1 mM phenylmethylsulfonyl fluoride, 0.1 mM benzethonium chloride, 10 mM EDTA and 20 KI aprotinin). The homogenate was centrifuged 8000× *g* for 10 min at 4 °C. The supernatant was frozen for further ELISA assay. CXCL1 and CCL3 levels were quantified by ELISA technique using commercial DuoSet kits from R&D Systems, according to the instructions of the manufacturer. Results were expressed in ρg of cytokines per ml of lung lysate (ρg/mL).

### 4.8. Virus Quantification (Plaque Assay)

Viral load was measured indirectly, through the quantification of viral hemagglutinin (HA) protein present in the middle lobe of the right lung by solid-phase sandwich ELISA using Influenza A H1N1 (A/Puerto Rico/8/1934) HA ELISA Pair Set (Sino Biological) under manufacturer’s protocol as described by [52].

### 4.9. Statistical analysis

All results were expressed as mean *±* standard error of the mean (SEM). Normalized data were analyzed by One-Way ANOVA with Tukey post-test, using the software GraphPad Prism 7.0. Statistical analysis for survival was performed using the Mantel–Cox test. Differences were considered significant at *p* < 0.05. Results are representative of three independent assays.

## Figures and Tables

**Table 1:** The anti-inflammatory effect of Maraviroc does not correlate with viral titer alterations.

H1N1	CsH1N1	CsH1N1 + Maraviroc
0.989 ± 0.195	1.059 ± 0.142	0.829 ± 0.129

Values of viral HA quantification are expressed as mean ± SEM in lung tissue samples from infected mice. Data are expressed as mean ± SEM from at least 6 mice per group.

## Data Availability

The data supporting the conclusions of this article will be made available by the authors under request, without undue reservation.

## Supplementary Materials

The following are available online at https://www.mdpi.com/1424-8247/14/7/620/s1, Figure S1: Mice weight loss at the end of the experiments (day 12). Figure S2: Gating strategy for the identification of neutrophils and macrophages in lung tissue. Figure S3: Expression of CCR5 in neutrophils and macrophages from lung tissue. Figure S4: Inflammatory exacerbation does not depend on CCR1 activation.
