# Supplementary material for: CCR5 Antagonist Maraviroc Inhibits Acute Exacerbation of Lung Inflammation Triggered by Influenza Virus in Cigarette Smoke-Exposed Mice
Source: Pharmaceuticals (Basel). 2021 Jun 28;14(7):620. doi: 10.3390/ph14070620 (PMC8308708; doi:10.3390/ph14070620)
Supplement: Supplementary file 1 [file pharmaceuticals-14-00620-s001.zip › pharmaceuticals-1228161-supplementary.pdf]

Supplementary Material (SM)

1. **Methods**

Mice

We assessed mice's welfare through observation of piloerection, respiratory rate, locomotion, feces alteration, water consumption, and amount of urine. No alterations in piloerection, respiratory rate, locomotion, and feces alteration were noted in our animals at the beginning of the assays.

2. Results

Supplementary Figure S1

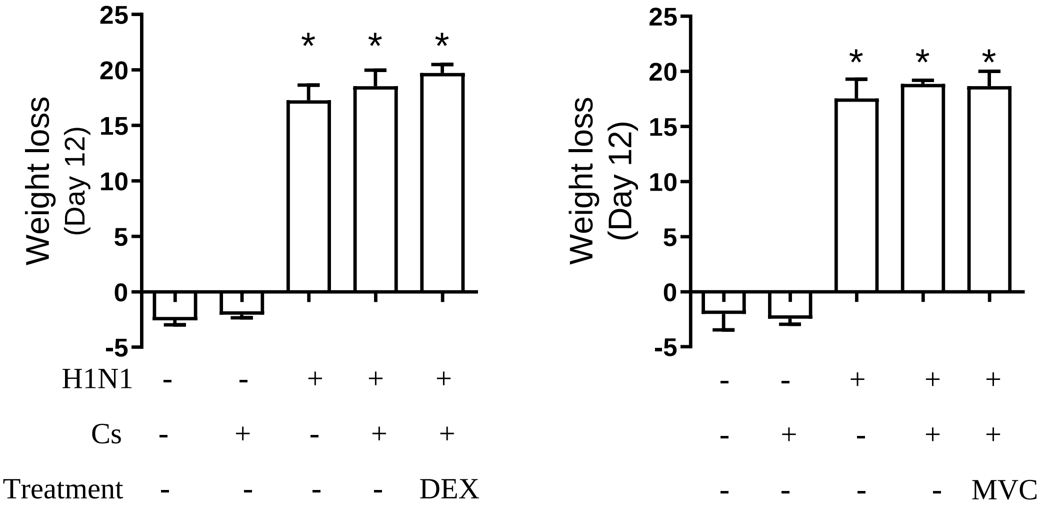

Supplementary Figure S1: Mice weight loss at the end of the experiments (day 12). Mice weight was assessed at the beginning of the *in vivo* protocol (see manuscript figure 1), day 0, and at the end of it, day 12, before being euthanized. Graphs show the % of weight lost at day 12 compared to the initial mice weight. Data are expressed as mean ± SEM from at least 6 mice per group. * for $p < 0.05$. Differences were statistically evaluated One-Way ANOVA with Tukey post-test.

Supplementary Figure S2

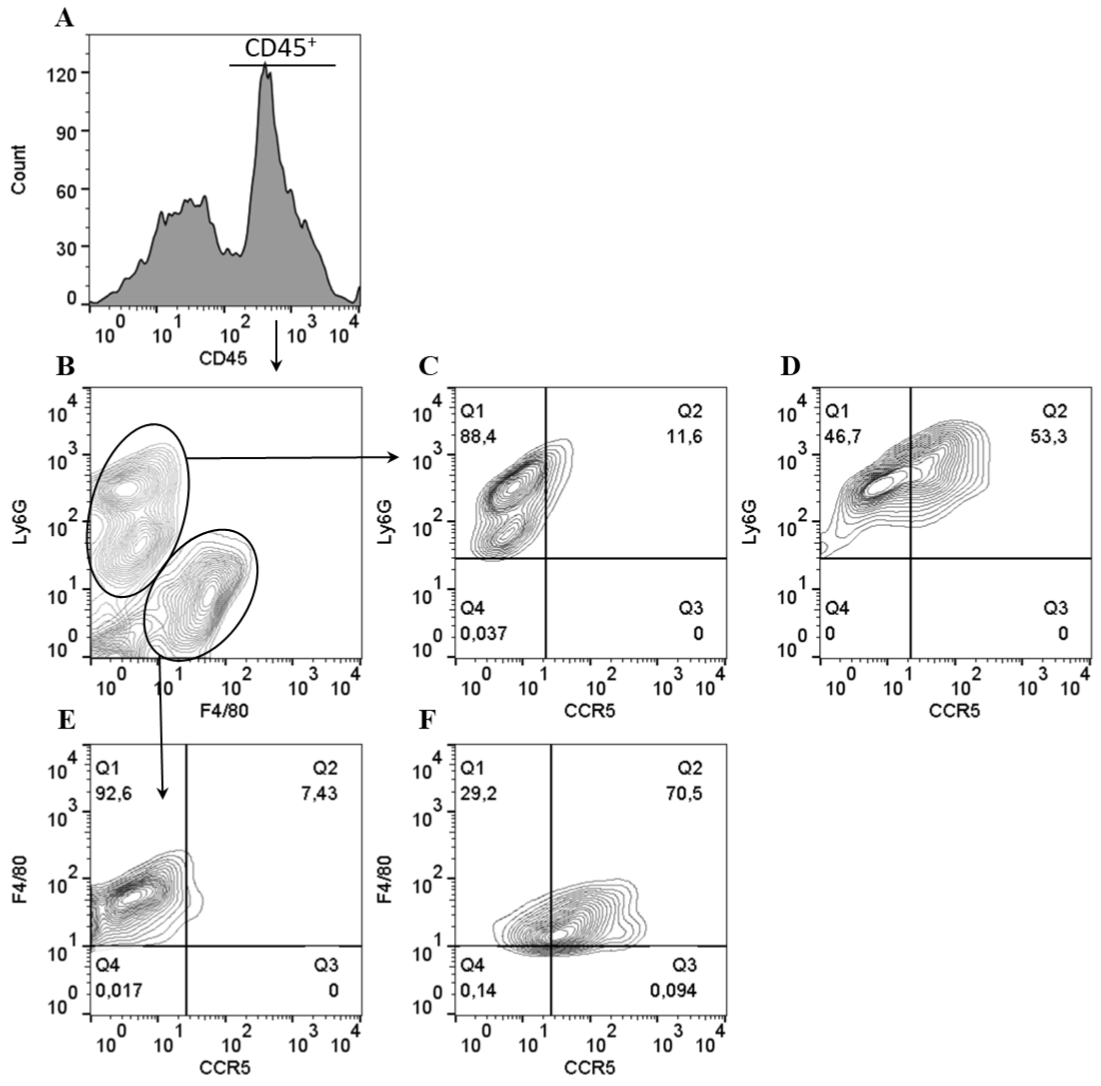

Supplementary Figure S2: Gating strategy for the identification of neutrophils and macrophages in lung tissue. Histogram and density plots of cells derived from lung tissue of mice. **(A)** Histogram showing $CD45^+$ cells in lung tissue. Dot plot showing **(B)** $Ly6G^+$ (Y axis) or $F4/80^+$ (X axis) cells present within the $CD45^+$ population. Dot plots showing the staining with isotype-matched **(C and E)** or anti-$CCR5^+$ **(D and F)** antibodies within $Ly6G^+$ or $F4/80^+$ sub-population in a CsH1N1 sample. All antibodies were validated by the producers and experimenters.

Supplementary Figure S3

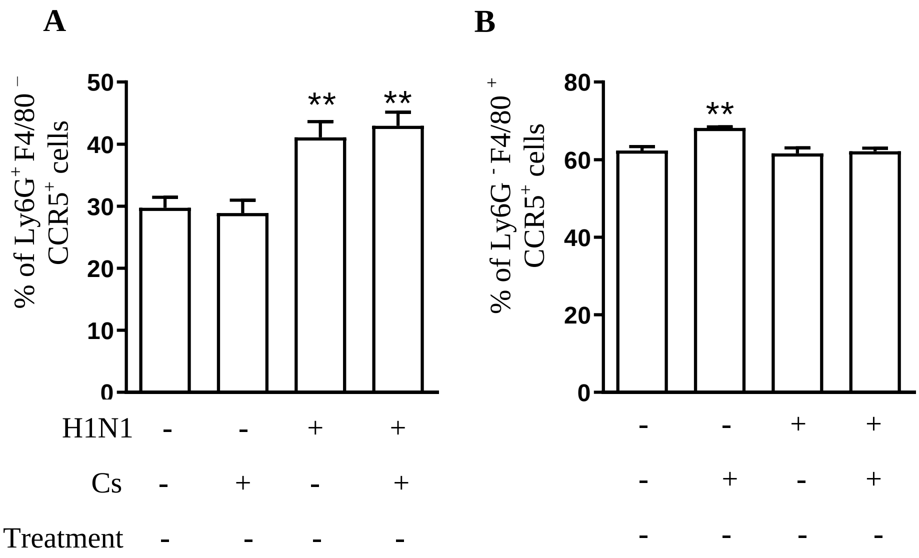

Supplementary Figure S3: Expression of CCR5 in neutrophils and macrophages from lung tissue. Percentage of (**A**) CCR5+ Neutrophils (CD45+/F4/80-/Ly6G+) and (**B**) CCR5+ Macrophages (CD45+/F4/80+/Ly6G-) in the lung tissue of mice exposed to Cs and/or infected with influenza A virus. Data are expressed as mean ±SEM from at least 6 mice per group. * for $p < 0.05$. Differences were statistically evaluated One-Way ANOVA with Tukey post-test.

Supplementary Figure S4

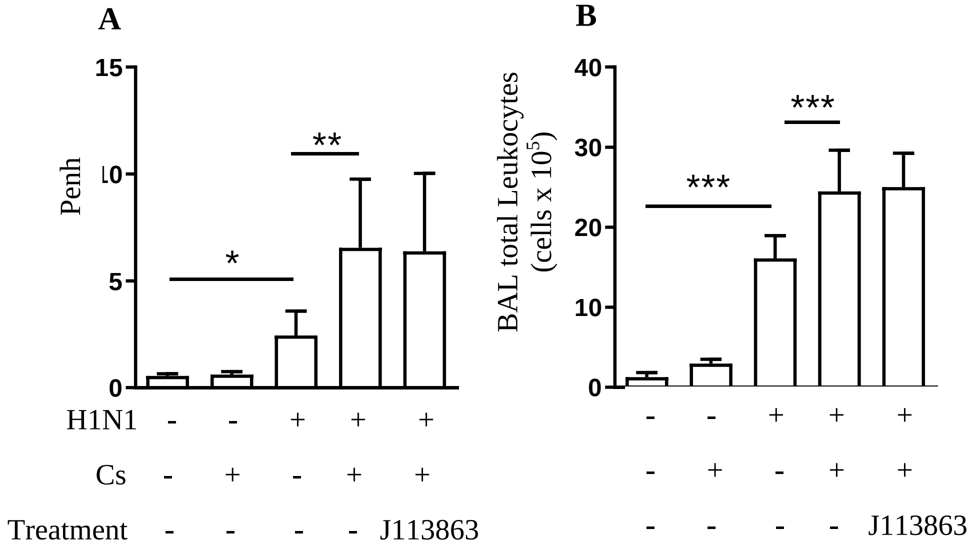

Supplementary Figure S4: Inflammatory exacerbation does not depend on CCR1 activation. Lack of effect of J113863 on the exacerbation of Penh elevation (**A**) and leukocyte infiltration (**B**) into the airways of mice exposed to Cs and infected with influenza A virus (CsH1N1). Data are expressed as mean ± SEM from at least 6 mice per group. * for $p < 0.05$. Differences were statistically evaluated One-Way ANOVA with Tukey post-test.
